# A Target-Less Vision-Based Displacement Sensor Based on Image Convex Hull Optimization for Measuring the Dynamic Response of Building Structures

**DOI:** 10.3390/s16122085

**Published:** 2016-12-08

**Authors:** Insub Choi, JunHee Kim, Donghyun Kim

**Affiliations:** Department of Architectural Engineering, Yonsei University, 50 Yonseiro, Seodaemun-gu, Seoul 120-749, Korea; insub@yonsei.ac.kr (I.C.); dhyun1227@yonsei.ac.kr (D.K.)

**Keywords:** vision-based displacement sensor, non-marker, image convex hull, dynamic characteristics, scaling factor map

## Abstract

Existing vision-based displacement sensors (VDSs) extract displacement data through changes in the movement of a target that is identified within the image using natural or artificial structure markers. A target-less vision-based displacement sensor (hereafter called “TVDS”) is proposed. It can extract displacement data without targets, which then serve as feature points in the image of the structure. The TVDS can extract and track the feature points without the target in the image through image convex hull optimization, which is done to adjust the threshold values and to optimize them so that they can have the same convex hull in every image frame and so that the center of the convex hull is the feature point. In addition, the pixel coordinates of the feature point can be converted to physical coordinates through a scaling factor map calculated based on the distance, angle, and focal length between the camera and target. The accuracy of the proposed scaling factor map was verified through an experiment in which the diameter of a circular marker was estimated. A white-noise excitation test was conducted, and the reliability of the displacement data obtained from the TVDS was analyzed by comparing the displacement data of the structure measured with a laser displacement sensor (LDS). The dynamic characteristics of the structure, such as the mode shape and natural frequency, were extracted using the obtained displacement data, and were compared with the numerical analysis results. TVDS yielded highly reliable displacement data and highly accurate dynamic characteristics, such as the natural frequency and mode shape of the structure. As the proposed TVDS can easily extract the displacement data even without artificial or natural markers, it has the advantage of extracting displacement data from any portion of the structure in the image.

## 1. Introduction

Building structures are exposed to repetitive or temporary external stimuli caused by disasters such as earthquakes and winds and to usage by the occupants of the building during the life cycle. These stimuli may cause small or large deformations of the structures. Because the outer wall of a building is the primary structure subjected to cyclic loads, experimental studies to identify the performance of the structure that forms the outer wall, and thus to propose a design equation, have actively been conducted of late [1,2,3]. If the behavior of a building structure can be accurately simulated using a mathematical model, the safety and serviceability of the structure can be evaluated precisely [4]. However, because the actual building structures include non-linear elements such as connections, boundary elements, material properties, and sectional properties, simulating such structures is difficult. Hence, a system identification (SI) technique of evaluating the status of the structure using field measurement data obtained through scale model experiments or monitoring of the actual structure is applied [5,6,7]; alternatively, a hybrid technique for improving the existing mathematical model by modeling the non-linear elements based on the measured data is used [8,9]. Therefore, obtaining data on the behavior of the structure is important because these data are directly or indirectly linked to the evaluation of the structure’s status.

The structure-related data to be measured can be divided into displacement and acceleration data. Because acceleration data contain global information about the structure, they exhibit low sensitivity in the estimation of local damages to the structure, and have the limitation that numerical errors occur when converting acceleration into displacement data [10]. The sensors for measuring the displacement of the structure can be generally classified into contact- and non-contact-type sensors. Contact-type sensors include a strain gauge or a linear variable differential transformer (LVDT). The data reliability of the contact-type sensor is known to be very high, but it has low applicability to actual buildings because it requires an additional apparatus, such as a fixed reference point, a power supply device, and a data collector [11]. The non-contact-type sensors, on the other hand, include a global positioning system (GPS) and a laser displacement sensor (LDS). These sensors have high applicability because they can be installed in a building structure more easily as compared to the contact-type sensors. The GPS, however, has a 5–10 mm error in the static state and a 10–20 mm error in the dynamic state, so the data accuracy is lower compared to that achievable with other sensors [12]. The LDS exhibits excellent performance, with a precision of approximately 0.2 mm [13], but the effective distance at which the equipment can obtain data is relatively shorter than that of other devices, and a fixed reference point is needed, as in the case of the contact-type sensors, when applied to an actual building. In this regard, there is a need for research into new sensors to overcome the limitations of the existing sensors, which cannot be easily applied to an actual building structure.

Since recently, studies were actively conducted on the non-contact-type vision-based displacement sensor (VDS) using images that can be easily obtained by a digital camera or a camcorder. The technical aspects of VDS related to the fields of electrical and electronic engineering [14,15,16,17] have shown much development, and VDS has been used in the civil engineering sector since the 1990s [18]. VDS was used in the monitoring system for measuring tensile force in a cable installed in a bridge and the reliability of the cable tensile force obtained from the VDS was high [19]. Feng and Feng [20] reported that the natural frequency of a short-span bridge could be measured by a VDS through field tests. Song et al. [21] experimentally showed that detection of damage in a cantilever beam was possible by processing displacement data using algorithms such as the wavelet-based damage detection algorithm. Hence, the VDS can be an alternative to the existing structural monitoring sensor to improve the applicability in the real field.

The initial VDS was used to attach the artificially produced marker to the structure, to analyze the movement of the marker in the image, and thus to extract the displacement data—This method is used widely today. Nogueira et al. [22] measured the displacement at the time of free vibration through the VDS after installing a circular marker at the end of a simple cantilever beam and extracted the natural frequency using the data obtained from the strain gage and the VDS, for comparison. The experimental results showed that the primary natural frequency of the simple beam obtained using the VDS was the same as the value obtained using the strain gage. Lee and Shinozuka [23,24] analyzed the natural frequency by measuring the real-time response of a bridge to which a circular marker plate was attached using the VDS and a laser vibrometer, and found that the VDS had sufficient accuracy to measure the dynamic characteristics of the structure. Ji [25] obtained displacement data by using the corner of a rectangular marker plate in the image. The developed VDS was applied to a cantilever beam and an actual bridge to compare the LVDT and the displacement data, and the developed VDS showed high precision. Park et al. [26] and Lee et al. [27] developed a method of measuring the deformation of a building structure; their method can be applied to the high-rise structure by installing cameras and marker plates in the building. They conducted an experiment by comparing the deformation in the structure with the LDS, which was a reference value; they found that the data reliability of the proposed method was very high. Choi et al. [28] proposed a VDS using a marker plate to measure the dynamic displacement of the structure. In an experiment using the shake table of a large-scale model, they experimentally proved that the dynamic displacement can be precisely measured using the VDS and marker plate. In addition, several researchers [29,30,31,32,33,34,35,36,37,38] have developed VDS devices for measuring the dynamic displacement of a structure, and verified the reliability of the displacement data obtained from the VDS by comparing these data with the data obtained by the existing sensor for an actual structure (mainly a bridge, which is a civil engineering structure) or the loading experiment of the scale model. In previous studies on VDS, the displacement data were extracted mostly by using a marker. The marker was used to provide the feature points for extracting the displacement data from the image. The VDS proposed by the above researchers has the limitation in that only the displacement data of the part with an attached marker are known, and if the marker falls off from the structure, those methods cannot be applied to obtain the displacement data using the VDS.

The purpose of installing the marker is not only to provide a feature point in the image, but also to calculate the scaling factor that converts the movement of the feature point from the pixel coordinates to the physical coordinates. In other words, if the marker were to be removed, it should be possible to calculate the scaling factor that converts the coordinates of the feature point without markers and a method of extracting the feature point without markers should be available. To solve the problem in which the existing VDS is accompanied by a marker, several works were conducted on extracting the displacement data [39,40,41] from some natural markers such as a rivet or a bolt hole of the structure in the image as feature points, and the dynamic characteristics of the structure were extracted precisely using the displacement data obtained from the image without artificial markers. In these works, a technique that was an extension of digital image correlation (DIC) was introduced to identify the feature points without artificial markers, and the scaling factor was calculated based on the focal length of the camera as well as the distance and angle between the camera and structure. Using this technique, the applicability of the proposed VDS to actual civil structures was proven, but the VDS cannot be used if there is no feature point that serves as a natural marker in the structure. In other words, the existing VDS extracts the displacement data from a natural or artificial feature point within the image depending on the use of a marker, but it has the limitation in that in the absence of a target that can be referred to as a feature point in the structure, the displacement data cannot be extracted. As the basic principle of the VDS transform the light information to the displacement data, external noise such as those attributed to the weather, ground vibration, and air heat haze can affect the accuracy of the displacement data obtained from the VDS. Xu et al. [42] reported that an error occurred in obtaining the displacement data from the VDS because of the unidentified object. Highly reliable displacement data can be obtained from VDS to consider various noise factors and to find the solutions prior to field test.

In this study, a target-less vision-based displacement sensor (TVDS) that can extract displacement data without a target such as artificial or natural marker within the image is proposed. The method of extracting the displacement data using the proposed TVDS is introduced, and the reliability of the displacement data obtained from TVDS was analyzed through experiments. In addition, the dynamic displacement data obtained from the TVDS and those obtained from the LDS compared through a white-noise test of a three-story scale model using a shake table, and the reliability of the structural dynamic measurement of the TVDS was analyzed by extracting the dynamic characteristics of the structure from the displacement data.

## 2. Target-Less Vision-Based Displacement Sensor (TVDS)

**Figure 1:**
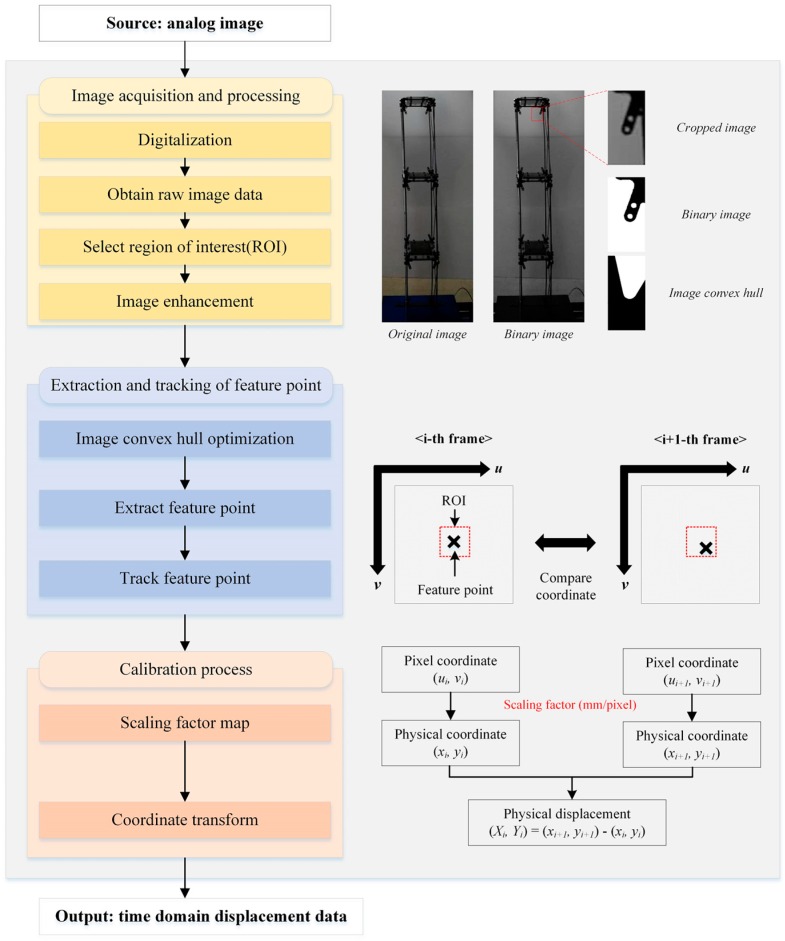
Displacement data acquisition diagram using the target-less vision-based displacement sensor.

Figure 1 illustrates the process of obtaining the displacement data from the image using the TVDS. The process largely consists of three steps: step (1) image acquisition and processing; step (2) extraction and tracking of the feature point; and step (3) calibration process. In step 1, an analog image of the actual structure is converted to a digitalized image through a camera. The converted image is imported into a computer, and a region of interest (ROI) is selected to improve the operation speed and to obtain the displacement data of the desired part. Thereafter, the noise in the image is removed through image enhancement for increasing the accuracy of the displacement data. In step 2, image binarization is conducted in the ROI of the selected image, and the feature point is extracted and tracked in the image without a target by using the image convex hull optimization technique for the obtained binary image. In step 3, the pixel coordinates of the feature point obtained in step 2 are converted into physical coordinates using the scaling factor map calculated based on the external environment between the structures and the camera and the location of the feature point within the image in order to obtain the time domain displacement data. The essential aspects of the proposed TVDS is the scaling factor map for converting the pixel coordinates to the physical coordinates and the method of extracting the feature point in the image without targets by using the image convex hull optimization.

### 2.1. Image Convex Hull Optimization

The results of several studies showed that highly reliable displacement data can be obtained from the image by using the existing VDS with a marker. There is a possibility, however, that the elimination of the marker will make it impossible to extract the displacement data. The alternative method of using natural markers such as the bolt hole of the structure also poses a limitation that the displacement data only for a specific part of the structure can be obtained. The TVDS proposed in this study can be said to be a VDS without a target because it is a method of obtaining the displacement data by extracting the feature point from the shape of the structure without artificial or natural markers referred as the target in the digitized image.

The key principle of the proposed TVDS is to find the feature point in the image using image convex hull optimization. The reason for proposing this method is that if parts such as the bolt hole or a corner of the structure in the image are found as feature points, the applicability of the vision-based sensor to the actual structure becomes lower. Image convex hull can be defined as a convex polygon that contains all the points in the binary image [43]. It has advantages that ensure the ease of extracting a feature point or a particular shape from an image, and its operation speed is faster [44]. There exist numerous convex hulls in a single image because the convex hull deals with the binary image determined depending on the threshold value ranging from 0 to 1. If the same convex hull is obtained from all the images by adjusting the threshold value in the continuous image, the structure in the image can be assumed to perform a rigid body motion, and the displacement data will be obtained with the centroid of the convex hull as the feature point. In this study, the feature point in the image was extracted through the optimization problem of the area of the image convex hull, as follows:

$$\begin{array}{l} \mathrm{Find}\;t_{i}\\ \mathrm{Min}\;\left|A{\left(t_{1}\right)}_{1}-A{\left(t_{i}\right)}_{i}\right|\\ s.t\;0\leq t_{i}\leq 1,\;i=2,\;3,\;\cdots ,\;n \end{array}$$

where, $t_{i}$ is the threshold value in i-th frame, $A_{i}$ is the area of image convex hull in i-th frame, and n is number of image frame.

**Figure 2:**
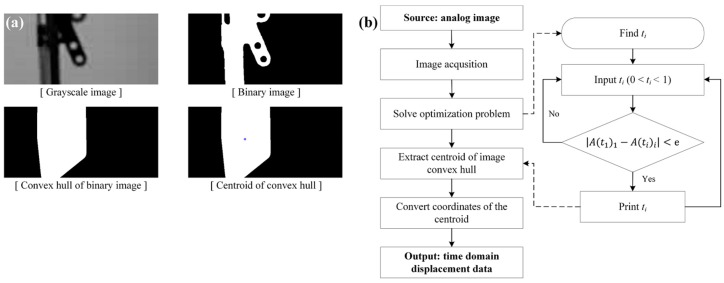
(**a**) Examples of the image convex hull; (**b**) Displacement extraction algorithm of the proposed TVDS.

Figure 2a shows examples of the feature point and image convex hull of a structure obtained through the process of the extraction and tracking of the feature point shown in Figure 1 in the structure images. In the selected ROI of the first frame, image binarization is conducted depending on the threshold value, and the image convex hull that contains all the white portions where the pixel value is 1 can be obtained in the binary image. The centroid is found in the obtained image convex hull, and the point is considered the feature point. Then, the convex hull optimized in the area of the first frame is found for each frame by adjusting the threshold value in each frame with respect to the area of the image convex hull calculated in the first frame in accordance with the flowchart in Figure 2b. Thereafter, the displacement data are extracted on a per-pixel basis from the changes in the center point of the image convex hull from all the image frames. To convert the extracted displacement data from pixel coordinates to physical coordinates, the scaling factor map proposed in Section 2.2 is used.

The proposed image convex hull optimization technique may be similar to the DIC technique, which does not require the target, however, the basic principle of the proposed technique differs from that of the DIC technique. The DIC technique provides a way to compare the distribution of the grayscale intensity level between the reference image and the deformed image. The correlation coefficient is defined in accordance with the grayscale intensity level between the two images, and by mapping each pixel, the value of the correlation coefficient is as found to be close to one. The one-by-one mapping of the DIC technique allows measurement of the strain filed, but the technique incurs a high computation cost, while the image convex hull optimization also uses the distribution of the grayscale intensity, the mapping process is not necessary to extract the displacement data from the image. The proposed technique is faster than the DIC technique owing to the omission of the one-by-one mapping, but it is difficult to extract the strain field. Although the proposed technique has a limitation for measuring the strain field, it can be said that the proposed technique is more suitable for measuring the displacement data than the DIC technique. Further, the DIC technique is sensitive to external light sources because the grayscale intensity level of the image is changed in accordance with the intensity of light illumination. The proposed technique extracts a feature point by comparing the shape of image convex hull determined in accordance with the threshold between the reference image and deformed image. Hence, the image noise resulting from the variance of light illumination does not significantly affect the accuracy of the proposed technique unlike in the case of the DIC technique.

As the method of finding the feature point using the image convex hull is to optimize the convex hull area of the first frame, it is available only when there is no deformation of the structure itself in the ROI of the image under the assumption of a plane motion. In the excitation test of the scale model using a shake table that was conducted in this study, no structural deformation or out-of-plane behavior caused by the torsion was observed, but there is a need to improve the algorithm of the proposed TVDS to be able to determine the critical displacement that leads to the collapse of the structure. Moreover, the image convex hull optimization has a limitation in extracting the feature point when large deformation occurs. Because the basic assumption of the image convex hull optimization technique in order to extract the shape of the structure is that the structure behave the rigid body motion (see [44]), the reliability of the displacement data will be decreased by the non-rigid body motion resulted from a large deformation of the structure. In other words, the image convex hull optimization has a limitation in extracting the feature point when a large deformation occurs.

### 2.2. Scaling Factor Map

The scaling factor is responsible for converting the pixel coordinates of the feature point to physical coordinates. The scaling factor (*SF*_1_) obtained through a marker is the ratio of the size of the actual marker to the size of the marker in the image, and can be easily obtained as follows:

(1) $$SF_{1}=\frac{D_{1}}{d_{1}}$$

where, *D*_1_ is the actual diameter of a maker (mm) and *d*_1_ is the diameter of a maker in the image plane (pixel).

If artificial marker is not used, the scaling factor (*SF*_2_) can be calculated from pixel pitch *R* of a camera sensor, focal length of *f*, and distance of *Z* between the camera and the target, as shown below:

(2) $$SF_{2}\left(cx,cy\right)=\frac{Z}{f}R$$

**Figure 3:**
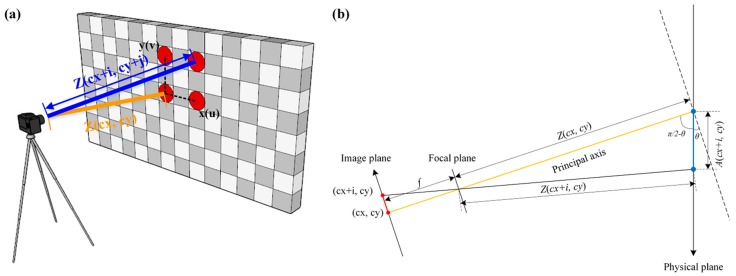
Relationship between the image plane and the physical plane. (**a**) 3D view; (**b**) Plane view.

In theory, *SF*_1_, which uses the size of the marker, and *SF*_2_, which uses the external conditions of the object and the camera, should be the same. However, experimental results showed that as the marker becomes more distant from the center of the image plane, a significant error occurs between the two scaling factors. Although Feng et al. [38] proposed the scaling factor determined by the distance and the angle between the camera and the target, and the angle for converting pixel coordinates to physical coordinates without a marker, an error occurred in the results obtained by comparing only the theoretical values, not the experimental ones in this study. Figure 3 is a graphical representation of the relationship between the camera and the target. It shows that the distance between the camera and the target obtained through camera changes depending on the position within the image (or the position of the actual target), whereas the focal length is constant. That is, as shown in Figure 3, the more distant a marker or an object from the center of the image plane, the greater is the distance *Z* from the camera, and therefore, adequate calibration process is required. The scaling factor map, in which the calculation of the scaling factor varies depending on the pixel coordinates in the image plane of the object can be obtained using the following equations:

(3) $$A\left(cx\pm i,cy\pm j\right)=\sqrt{(i\times SF_{2}{\left(cx\pm i\mp 1,cy\right)}^{2}+(j\times SF_{2}{\left(cx,cy\pm j\mp 1\right)}^{2}}$$

(4) $$Z\left(cx\pm i,cy\pm j\right)=\sqrt{Z{\left(cx,cy\right)}^{2}+A{\left(cx\pm i,cy\pm j\right)}^{2}-2Z\left(cx,cy\right)A\left(cx\pm i,cy\pm j\right)\cos\left(\theta \right)}$$

(5) $$SFM\left(cx\pm i,cy\pm j\right)=\frac{Z\left(cx\pm i,cy\pm j\right)}{f}R$$

where, A is the distance, *cx* and *cy* is center pixel of image plane, and SFM is scaling factor map.

The image correction method is needed to obtain reliable displacement data from the image convex hull optimization in the case of short focal length and small distance between the camera and structure. Generally, affine transformation or homography transformation should be used to compensate the distorted image. As we assumed that the structure has no target, it is hard to use the typical image correction methods using the target points. The image correction was automatically performed by the coordinate transformation using the proposed scaling factor map, because the scaling factors varied in accordance with the location of the feature point obtained from the image convex hull optimization without the target as well as the external environments such as the focal length and distance. At the end of this section, the results of the marker plate test to estimate the actual size of marker are summarized in accordance with the distance and angle, which may cause image distortion.

**Figure 4:**
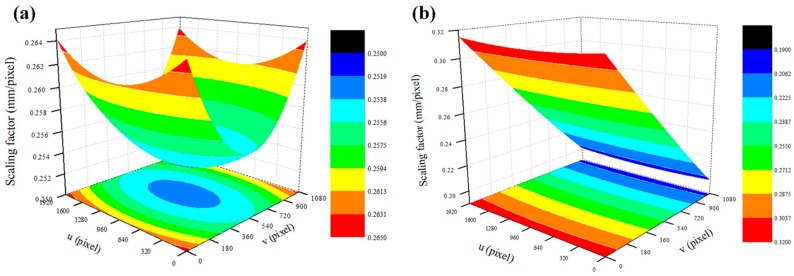
Scaling factor map according to the distance, angle, and focal length. (**a**) Distance = 930 mm, angle = 0, focal length = 45 mm; (**b**) Distance = 930 mm, angle = 60°, focal length = 45 mm.

The camera used in this study was a Nikon D5500 (full-HD, 60 fps), and a wide-angle lens of F-mount with an 18–55 mm focal length. At full HD (1920 × 1080 pixels), the *cx* and *cy* values are 960 and 540, respectively. The R value refers to the size of the actual sensor that one pixel occupies in the image plane as a pixel pitch, and in video shooting with Nikon D5500, the *R* value is 12.24 μm. Figure 4 shows a scaling factor map in the same external environment, in which only the angle between the camera and the target is different. As shown in Figure 4, the pixel coordinates of the feature point can be converted to physical coordinates through the scaling factor map, which is calculated differently depending on the pixel coordinates of the feature point.

**Figure 5:**
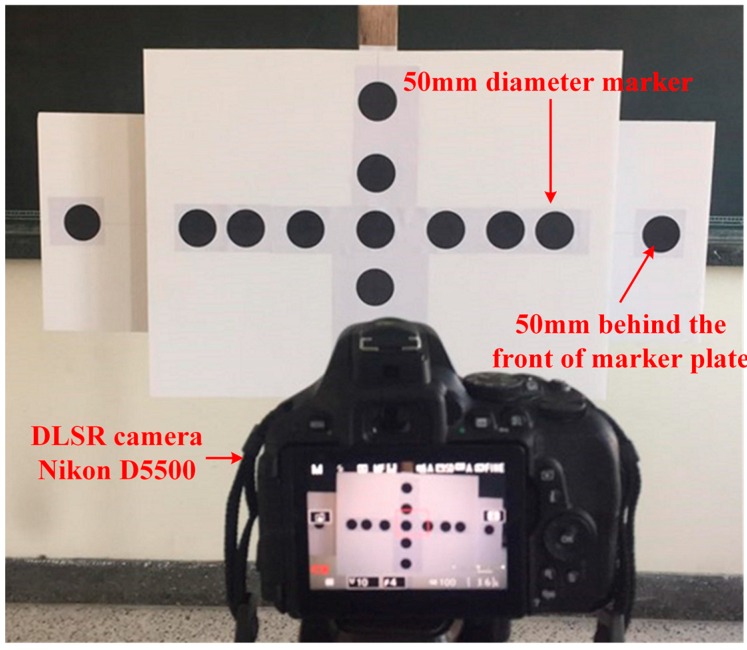
Scaling factor map validation test using a 50-mm-diameter marker plate and a commercial camera.

In order to verify the scaling factor map that is related to the data accuracy of the TVDS, a marker plate with a thirteen of 50 mm-diameter markers was installed in front of a camera as shown in Figure 5, and the *SF*_1_ and *SF*_2_ values obtained from the size of marker and the scaling factor map, respectively, were compared. The markers at both ends of the right and left sides were located 50 mm behind from the front markers, and for the experiment, the distance between the camera and the marker was increased by 930 mm, up to 930–3720 mm, and the angle between the camera and the marker plate was increased by 10°, up to 0°–60°. The distance and the angle are the variables to affect the image distortion, which make difficult to estimate the actual size of marker using the *SF*_2_ obtained from the scaling factor map.

**Figure 6:**
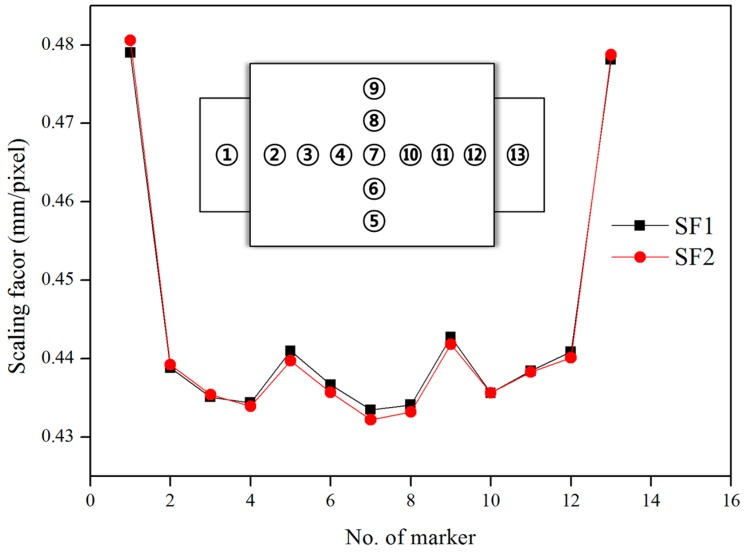
Comparison of the scaling factors calculated by the marker and the scaling factor map, respectively.

**Figure 7:**
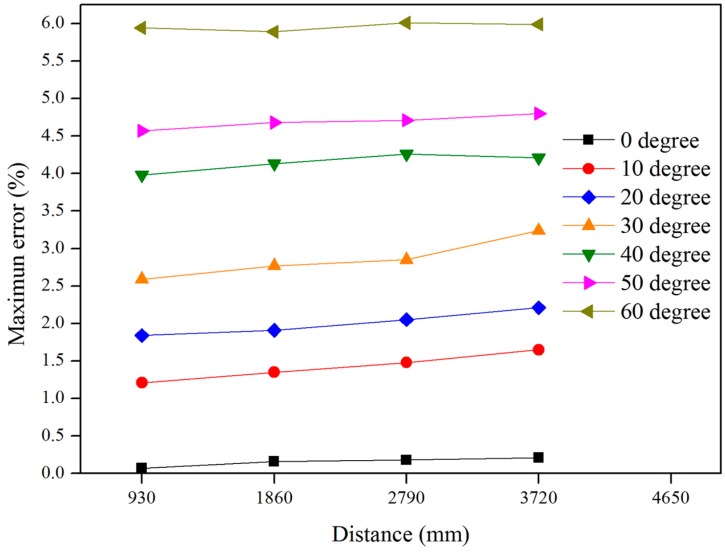
Maximum scaling factor error between SF_1_ and SF_2_ in accordance with the distance and the angle.

Figure 6 shows a graph that compares the difference between the *SF*_1_ and *SF*_2_ values at distance = 930 mm, angle = 0, and focal length = 45 mm when the error was approximately 0.36%, and the diameter difference of the marker was 0.09 mm. These results imply that the image distortion was resolved through the scaling factor map despite the short distance. Figure 7 is a graph showing the maximum error of *SF*_2_ according to the distance and the angle, and it can be seen that the error in the distance was low, but it increased greatly with an increase in the angle. When the angle was constant, the difference in diameter with the actual marker with an increase in the distance ranged from 0.03 to 0.11 mm. This indicates that the error is not governed by the distance. When the distance was constant, however, the diameter difference with the actual marker ranged from 1.41 to 1.47 mm, showing a larger difference compared to the distance. The results of the marker plate test showed that the image distortion caused by the angle between the camera and the object plane is significantly effect on the data accuracy, and the proposed scaling factor has the limitation that compensation for the distorted image is necessary when the angle is larger than 30°. Based on these results, the angle between the camera and the structure was reduced to less than 10° in order to reduce the error due to external factors.

## 3. Experimental Test Set-Up

**Figure 8:**
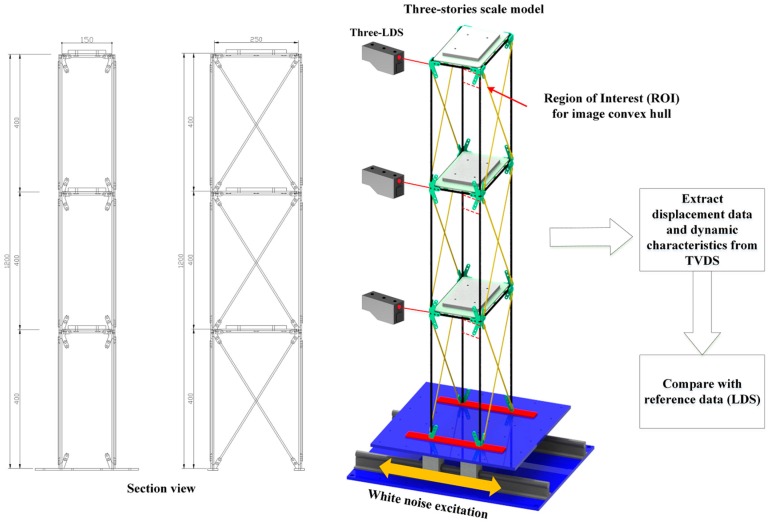
Dimensions of the scaled model and experimental set-up of scaled model white noise excitation test using shake table (unit: mm).

A three-story scaled model test was conducted using a shake table (QuanserShake Table II, Quanser Consulting Inc., Markham, ON, Canada) in order to measure the dynamic displacement through the proposed TVDS. The dynamic displacement of the scaled model was measured using the TVDS and LDS. The displacement data obtained from the LDS were used as reference data to verify the displacement data reliability of the TVDS. Figure 8 shows the details of the scaled model and the shake table excitation experiment.

Because the proposed TVDS employs a camera and extracts the shape of the structure by the image convex hull optimization to measure the displacement of the scaled model, the reliability of the displacement data will decrease in the event of out-of-plane behavior. The displacement data could be extracted using a camera despite the out-of-plane behavior if homography transformation is applied and the camera is installed at an angle to the moving plane of the structure. However, the image convex hull optimization used in this study has a limitation in extracting the points required for the transformation. Hence, an X-shape brace was installed in a longitudinal direction of the scaled model to prevent the out-of-plane behavior of the scaled model. The member and materials properties of the scaled model are summarized in Table 1.

**Figure 9:**
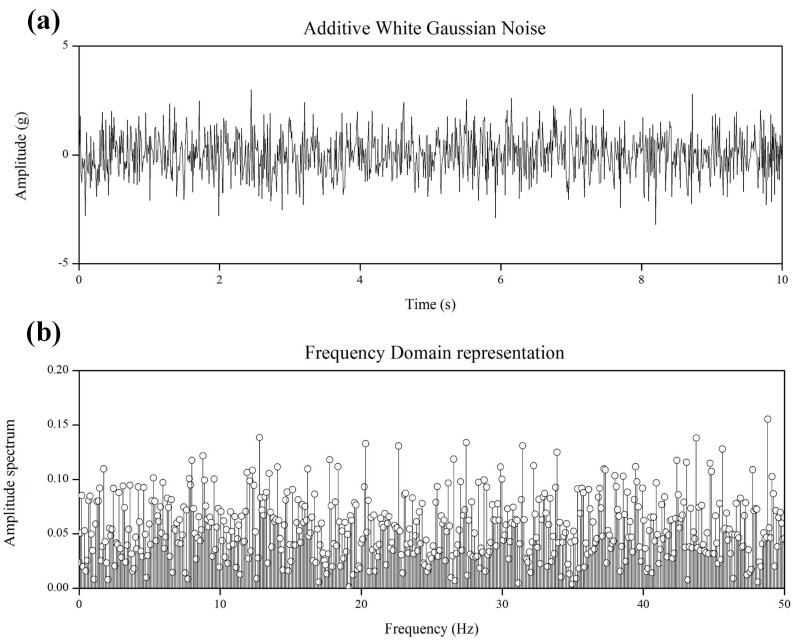
50 Hz-bandwidth white noise. (**a**) Time domain acceleration data; (**b**) Frequency domain representation data.

The weight of the mass plate of each floor was approximately 2.0 kg, and the total mass of the scaled model was approximately 9.08 kg. To facilitate the detection of the dynamic characteristics of the structure from the obtained displacement data, a load was applied to the specimen with a 50-Hz-bandwidth white noise, as shown in Figure 9. White noise was generated using MATLAB, and the white noise generated up to 0–50 Hz included a total of 513 frequency components at 0.0977 Hz intervals.

The TVDS extracted feature points by optimizing the area of the image convex hull obtained on the first frame, with the right side of the scaled model as the ROI. The distance between the camera and the scaled model was 1300 mm, the focal length was 18 mm, and the angle was 3°. From these external environments, the calculated scaling factor in the center position of the image was found to be 0.8840 mm/pixel. The image of the scaled model was captured with the Nikon camera at 1920 × 1080 (2.0 megapixels) and 60 fps.

## 4. Test Results and Discussion

This section describes the analysis of the reliability of the dynamic displacement data obtained from the TVDS. The displacement data based on vision were directly compared with the reference data of LDS. In addition, the natural frequency and mode shape of the scaled model were extracted using the obtained displacement data, and a comparison with the analytical values was conducted using a commercial program named “MIDAS-Gen”.

### 4.1. Comparison of Dynamic Displacement Data

**Figure 10:**
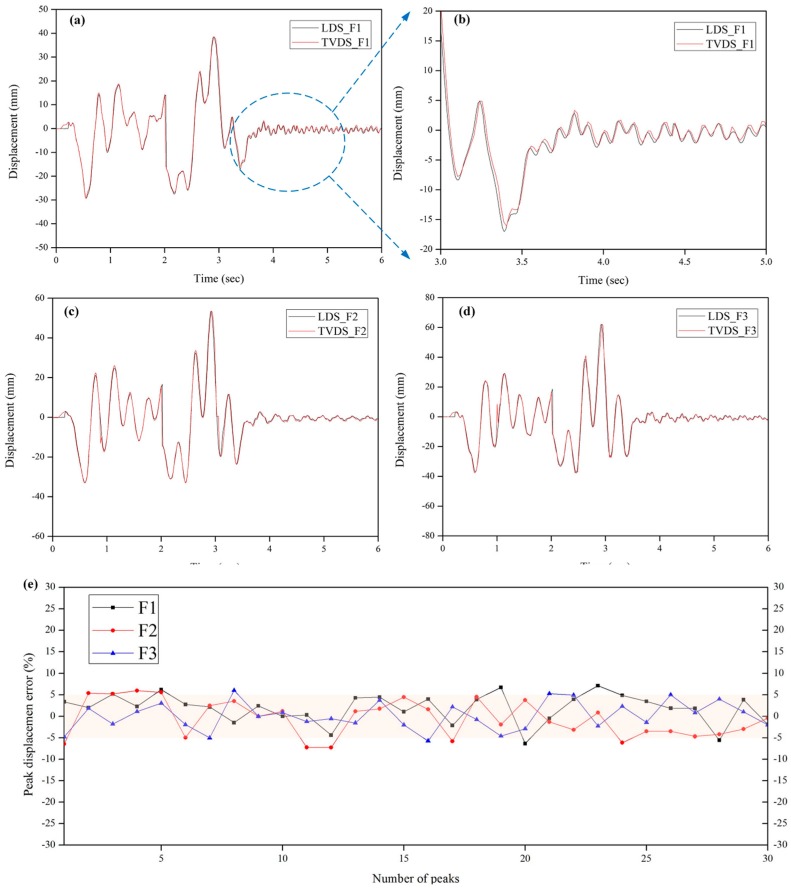
Comparison of dynamic displacement data between VDS and LDS according to the floor level. (**a**) 1st floor; (**b**) detail comparison of the 1st floor; (**c**) 2nd floor; (**d**) 3rd floor; (**e**) peak displacement error.

A direct comparison of the dynamic displacement data between LDS and TVDS was performed in order to analyze the reliability of dynamic displacement data obtained from the TVDS. Figure 10 shows a time-displacement graph that compares the displacement data obtained from the TVDS and LDS of the scaled model to which white noise was applied. With respect to the size and period of the data, the displacement data of the scaled model obtained from the TVDS was in good agreement with the displacement data obtained from the LDS. In addition, it was found that the TVDS can precisely measure not only a relatively large displacement of 40 to 70 mm in the structure at the time of white noise excitation, but also a relatively small displacement of less than 4 mm occurring during free vibration after excitation. Figure 10b shows that the graph obtained by expanding the displacement data measured at 3 to 5 s on the 1st floor. As can be seen in the Figure 10b, some differences occurred in the maximum and minimum values between the two measured data, but the vibration period of the scaled model matched well.

Using the scaling factor map calculated from the focal length, angle, and distance of the scaled model and the camera, the pixel coordinates could be converted to physical coordinates. The scaling factor of the central portion of the image was 0.8840 mm/pixel, and that at the end corner of the image was 1.1508 mm/pixel. In addition, as one pixel was divided into five pixels (five-subpixel level) in the original image when processing the image data, the resolution of the TVDS was 0.1768 to 0.2302 mm/pixel. If the subpixel level will be increased, the resolution of the TVDS will be lowered, and thus, the accuracy of the sensor can be enhanced. However, there are disadvantages to increasing the subpixel level because the time needed for extracting displacement data from the image will increase and the pixel information of the original image can be distorted. Accordingly, an appropriately adjusted subpixel level is necessary. Although the one by one comparison of the two displacement data is a good way to reveal the true difference between both data, it is difficult to directly compare the two data sets because of the different sampling rates of the two sensors. Hence, the differences in the peak displacements of LDS and TVDS were compared, and the peak error represented the peak difference between them was calculated from Equation (6). Figure 10e shows the peak error between LDS and TVDS in accordance with the floor level. The maximum peak error is error is 7.16% on the 1st floor, 5.97% on the 2nd floor, and 6.06% on the 3rd floor, and the absolute mean value of peak error is 3.36% on the 1st floor, 3.70% on the 2nd floor, and 2.69% on the 3rd floor. The aforementioned experiment, as shown in Figure 10b,e, revealed that sufficient sensor resolution can be secured for the displacement data obtained using the TVDS, even when compared to the LDS with a 0.01 mm resolution:

(6) $$Peak\;error\;\left(\%\right)=\frac{TVDS-LDS}{LDS}\times 100\%$$

where, TVDS and LDS are the peak displacement obtained each measuring methods.

To quantitatively analyze the reliability of the displacement data obtained from the TVDS, it was numerically compared with the LDS through root mean square (RMS) analysis, and the results are summarized in Table 2. The RMS difference between the LDS and the TVDS was 0.22% on the 1st floor, 2.85% on the 2nd floor, and 1.12% on the 3rd floor. Although the biggest error occurred on the 2nd floor, the numerical error between the two data did not exceed 3%. This indicates that even though there is no natural or artificial marker that can be referred to as a target in the structure, the use of the proposed TVDS makes it possible to acquire the dynamic displacement with high reliability.

### 4.2. Dynamic Characteristics Extracted from VDS

**Figure 11:**
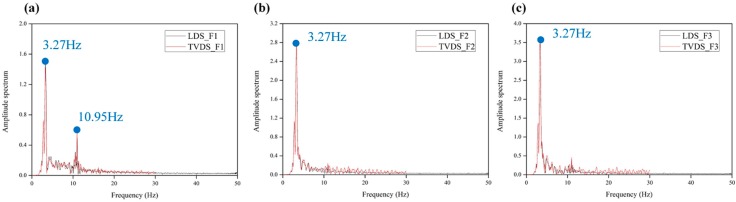
Comparison of natural frequencies obtained from the TVDS and LDS according to the floor level. (**a**) 1st floor; (**b**) 2nd floor; (**c**) 3rd floor.

If the dynamic characteristics of the structure can be extracted using the obtained displacement data, they can be used for system identification or damage assessment, and therefore, the analysis of the reliability of the obtained dynamic characteristics is important for verifying sensor performance. The dynamic characteristics of the structure, such as the mode shape and natural frequency, were extracted by converting the time domain displacement data to frequency domain data. Figure 11 shows a graph that compares the natural frequencies obtained by the LDS and TVDS. Both sensors yielded 3.27 Hz as the primary natural frequency, and the value of the amplitude was also similar. The secondary natural frequency was not obtained noticeably in the second or third floor data, but it was 10.95 Hz in the displacement data of the first floor. The reason for the failure to obtain more than the third natural frequencies definitely in both the TVDS and the LDS from the second and third displacement data is that it is easy to obtain the data of a low-frequency domain owing to the characteristics of the displacement data, and the sampling rates of 1.67 ms (TVDS) and 1 ms (LDS) are too high for obtaining the natural frequencies of more than 10 Hz of the scaled model.

To verify the accuracy of the dynamic characteristics obtained from the displacement data, the natural frequencies were compared after modeling the scale model using the numerical analysis program MIDAS-Gen. The results are summarized in Table 3. The error between the natural frequency obtained using the numerical analysis model and that obtained based on the experiment results was less than 1%. Further, in the experiment, both the LDS and TVDS failed to obtain the third natural frequencies. It can be seen that the TVDS can extract the primary and secondary natural frequencies almost precisely and thus secure a performance identical to that of the LDS in this experiment. The comparison of the experiment and analytical data revealed that the natural frequency of the scaled model obtained by the numerical analysis model was higher than that of the experiment results. This is because the stiffness of the beam-to-column connection of the actual scaled model was idealized as a rigid connection in the numerical analysis model.

**Figure 12:**
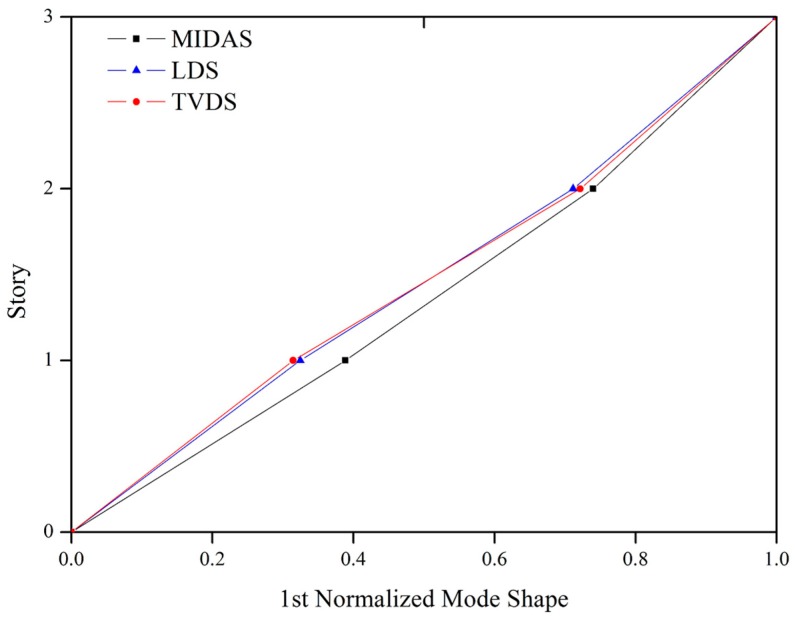
Comparison of the analytical and experimental mode shapes of the scaled model.

Figure 12 shows a comparison of the numerical and experimental mode shapes of the scale model.

The experimental mode shapes obtained by both the LDS and the TVDS were similar, and the results only differed slightly from the mode shape obtained numerically. The reason for the difference is that the connection stiffness of the numerical model and that of the actual scaled model are different; this is similar to the case of the differences in the natural frequency. It was experimentally confirmed that the use of the proposed TVDS makes it possible to precisely obtain the mode shape and natural frequency of the structure, and the accuracy of the obtained data was verified through comparison with the analysis results.

## 5. Conclusions

In this study, a target-less vision-based displacement sensor (TVDS) was developed to measure the displacement data of the structures without any target such as an artificial or a natural marker in the image. The core technologies of the TVDS are the image convex hull optimization algorithm for extracting the feature points without the artificial markers attached to the structure or natural markers such as a bolt hole in the structure, and a scaling factor map that converts the pixel coordinates of the extracted feature point to physical coordinates using the external environment between the camera and the structures. The reliability of the displacement data obtained by the TVDS was verified experimentally through an excitation test for a three-story scaled model, and through a marker plate test. The study results are summarized as follows:

(1) In the TVDS, the scaling factor map is used to convert the pixel coordinates of the feature point obtained from the image convex hull optimization algorithm to physical coordinates. Unlike the existing scaling factor obtained from the diameter of the marker, the scaling factor map can be calculated from the distance between the camera and the marker, the angle, and the focal length. The factor is calculated in accordance with the pixel coordinates of the feature point on the image plane. In the marker plate experiment designed for estimating the diameter of a 50-mm-diameter marker, the error depending on the distance was up to 2%, and that depending on the angle was up to 6%. That is, the error of the estimation by the proposed TVDS was caused to a greater extent by the angle than by the distance. Hence, it is necessary to measure the angle between the camera and the target and set it within 10° at the maximum in actual applications.
(2) The white-noise excitation test of the three-story scaled model revealed that as the TVDS does not require artificial or natural markers, it can provide the displacement data of three floors from one image. In addition, the difference between the RMS values of the displacement data obtained by the TVDS and the reference data LDS was 0.22%–2.85%, and therefore, it was experimentally verified that the TVDS can perform multiple extraction of highly reliable displacement data with a single camera.
(3) To determine if TVDS is a suitable sensor for monitoring a structure, the dynamic characteristics of the structure, such as the mode shape and natural frequency, were extracted using the displacement data obtained by the TVDS. The primary natural frequency values of the scale model obtained by the TVDS and the LDS were identical (3.27 Hz), showing only a 0.82% difference from the primary natural frequency obtained using the numerical analysis model (3.31 Hz). In addition, the mode shape obtained by the TVDS was very similar to the experimental mode shape obtained by the LDS and the analytical mode shape obtained based on the numerical analysis results. In other words, as the displacement data and the dynamic behavior of a structure can be precisely measured through the proposed TVDS, the TVDS is considered a suitable sensor for monitoring a building structure.

The proposed TVDS was shown to be capable of measuring the displacement of the desired part of the structures precisely by using the image convex hull optimization algorithm and scaling factor map. It could also measure the dynamic characteristics of the structure, such as the mode shape or natural frequency. Therefore, as the use of the TVDS makes it possible to obtain the displacement data of a structure without using any target, which is required by the existing vision-based displacement sensor (VDS), the TVDS is expected to become more widely used in the structure monitoring field in the future.

## Figures and Tables

**Table 1:** Member properties of the scaled model.

Member	Material	Section Size (mm)
Column	SS400	5 × 5
Girder		4 × 6
Brace		4 × 6
Slab	Acrylic	5t

**Table 2:** Displacement data reliability results on the scaled-model excitation test.

Story	RMS (mm)		Error
	LDS	TVDS	
1	8.0810	8.0988	0.22%
2	9.7498	10.0356	2.85%
3	10.9949	11.1191	1.12%

**Table 3:** Experimental and analytical natural frequencies of the scaled model.

No. of Mode	Analytical Model (Hz)	Test Result (Hz)	
		LDS	TVDS
1	3.31	3.27 (0.82%)	3.27 (0.82%)
2	11.08	10.95 (0.94%)	10.95 (0.94%)
3	17.57	-	-
